# Editorial: Epigenomics implication for economic traits in domestic animals

**DOI:** 10.3389/fgene.2023.1252640

**Published:** 2023-07-17

**Authors:** Chunfang Zhao, Bo Han, Guoqiang Yi, Sihua Jin, Zebin Zhang, Shenghe Li

**Affiliations:** ^1^ College of Animal Science, Anhui Science and Technology University, Chuzhou, Anhui, China; ^2^ Anhui Province Key Laboratory of Animal Nutritional Regulation and Health, Chuzhou, Anhui, China; ^3^ Department of Animal Genetics and Breeding, Key Laboratory of Animal Genetics, Breeding and Reproduction of Ministry of Agriculture and Rural Affairs, National Engineering Laboratory for Animal Breeding, College of Animal Science and Technology, China Agricultural University, Beijing, China; ^4^ Shenzhen Branch, Guangdong Laboratory of Lingnan Modern Agriculture, Genome Analysis Laboratory of the Ministry of Agriculture and Rural Affairs, Agricultural Genomics Institute at Shenzhen, Chinese Academy of Agricultural Sciences, Shenzhen, China; ^5^ College of Animal Science and Technology, Anhui Agricultural University, Hefei, China; ^6^ Division of Plant Ecology and Evolution, Department of Ecology and Genetics, Evolutionary Biology Centre, Uppsala University, Uppsala, Sweden

**Keywords:** domestic animals, epigenomics modification, economic traits, ancient DNA, genetic markers

Disease resistance, reproductive performance, lactation performance, meat production, egg production, down production, meat quality, egg quality, and other traits are crucial for the economic performance of the livestock farming industry and the quality of livestock products. Epigenetic modifications have a significant impact on the expression of these traits, including DNA methylation, histone modifications, chromatin remodelling, and non-coding RNAs that regulate gene expression. Epigenetics is involved in various biological processes, including embryonic development, tissue differentiation, and tumourigenesis. Environmental stress can influence epigenetic modifications, which are stored in the genome through chromatin modifications and high-level structures. Factors such as nutrition, feeding practices, and environmental factors can influence epigenetic modifications. As biotechnology advances, the development of experimental and analytical methods in epigenetic modifications becomes increasingly important.

In this Research Topic, we cover genetic markers associated with the economic traits of different livestock breeds, various epigenetic modifications that affect livestock economic traits, and the applications of different epigenetic modifications in domestic animals.

## Classification of epigenetic modifications

Epigenetic modifications such as DNA methylation, histone modifications, and non-coding RNAs can affect the expression patterns of genes related to economic traits in livestock, thereby exerting significant effects on livestock performance. For example, Liu et al. found that the expression level of miR-499-5p was significantly positively correlated with the content of skeletal muscle type I fibers and significantly negatively correlated with the content of type IIB fibers by comparing the gene expression patterns of different types of chicken muscle fibers. This study suggests that non-coding RNA is a type of epigenetic modification that exists in different quality chicken breeds with differential expression levels. In addition, Caiye et al. conducted a genome-wide DNA methylation association analysis on three breeds of sheep, Lanzhou Big-Tail Sheep, Altay Sheep, and Tibetan Sheep, and identified differential methylated regions or sites, revealing the differential DNA methylation modifications during tail formation in different sheep breeds. Meanwhile, Erven et al. used interpolation with low coverage ancient *Sus scrofa* data to detect genetic mutations and polymorphisms resulting from the evolution of ancient DNA (aDNA), and inferred errors in identifying or over-representing ancestral components and selective features in the genotype. These findings shed light on the mechanisms behind the emergence and spread of early animal husbandry in Europe and the Near East during the entire Neolithic era, based on genetic variation.

## Genetic markers associated with economic traits in domestic animals

Focusing on genetic markers associated with novel economic traits in livestock can help to screen for individuals carrying superior genetic markers, thereby contributing to the productivity and economic efficiency of livestock. Wen et al. compared the spectra of extrachromosomal circular DNA (eccDNA) and extrachromosomal DNA (ecDNA) between Wei pigs (WP) and Large White pigs (LP), and found that the abundance of eccDNA was lower, while the abundance of ecDNA was higher in WP compared to LP. Although eccDNA and ecDNA are common in both WP and LP, their sizes are large enough to carry one or more partial or complete genes, making them a potential new genetic marker for selecting high-quality breeds. A study by Cao et al. found that acyl-CoA synthetase long-chain family member 1 (ACSL1) has two different transcripts, namely, ACSL1-a and ACSL1-b. Their research comprehensively elucidated the crucial role of the ACSL1 gene in triglyceride synthesis, which may provide molecular markers for screening high-quality lamb meat. Interestingly, because Alvarenga et al. found that temperament is highly heritable and polygenic, with favorable genetic correlations with other relevant traits, so the temperament of cattle has been regarded as a key breeding goal by farmers.

## The relationship between epigenomics and economic traits

Epigenetic modifications play an important role in the economic traits of domestic animals, and in-depth research on the relationship between epigenetic modifications and economic traits can provide theoretical and practical guidance for domestic animals breeding and improvement of economic traits. Caiye et al. identified 68,603 differentially methylated regions (DMCs) and 75 differentially methylated genes (DMGs) associated with these DMCs, of which the DMGs (NFATC4, LPIN2, MGAT2, and MAT2B) are mainly involved in the process of fat metabolism, which providing the first evidence for the relationship between tail fat deposition in local sheep and epigenetics. Two studies by Zheng et al. revealed the dynamic changes in protein expression during the development of the rumen epithelial cell, identifying the major proteins involved in rumen epithelial cell development. These studies also provided preliminary evidence for the importance of post-translational modifications of histones in the development of the rumen epithelial cell. It is necessary to screen epigenetic modifications that regulated DMGs associated with economic traits for further application in breeding.

## Prospects for improving economic traits based on epigenetic modifications

Using epigenetic modification techniques, the genome can be directly edited and regulated, allowing for rapid changes in the economic traits in domestic animals. Wang et al. reported that transgenic mice were generated, which exhibit skin-specific overexpression of ovarian β-catenin. The excess expression of β-catenin promotes the transition of hair follicles from the growth phase to the maturation phase, thereby increasing hair follicle density. This provides a new approach for using transgenic technology to improve economic traits in domestic animals. Different economic traits may be associated with different epigenetic modifications. By regulating these epigenetic modifications, simultaneous improvement of multiple traits can be achieved. In fact, Liu et al. found that non-coding RNAs are associated with chicken meat quality, while Caiye et al. suggested that DNA methylation is related to the formation of sheep tail fat, which confirms the association between different traits and different genetic markers. The prospects for improving economic traits based on epigenetic modifications are broad, but breeding methods based on epigenetic modifications are still in the developmental stage and require further research and validation.

In summary, this Research Topic collected a large amount of research evidence confirming the close relationship between epigenetic modifications and economic traits in domestic animals, while proposing new breeding ideas for improving animal economic benefits.

